# Reliability and validity of ten consumer activity trackers

**DOI:** 10.1186/s13102-015-0018-5

**Published:** 2015-10-12

**Authors:** Thea J. M. Kooiman, Manon L. Dontje, Siska R. Sprenger, Wim P. Krijnen, Cees P. van der Schans, Martijn de Groot

**Affiliations:** 1 Research group Healthy ageing, Allied health care and Nursing, Hanze University of Applied Sciences, Groningen, The Netherlands; 2 CBO Groningen: Center for Physical Activity and Research, Groningen, The Netherlands; 3 Quantified Self Institute, Hanze University of Applied Sciences, Groningen, The Netherlands

**Keywords:** Accelerometry, Activity trackers, Validation study, Reliability, Free-living

## Abstract

**Background:**

Activity trackers can potentially stimulate users to increase their physical activity behavior. The aim of this study was to examine the reliability and validity of ten consumer activity trackers for measuring step count in both laboratory and free-living conditions.

**Method:**

Healthy adult volunteers (*n* = 33) walked twice on a treadmill (4.8 km/h) for 30 min while wearing ten different activity trackers (i.e. Lumoback, Fitbit Flex, Jawbone Up, Nike+ Fuelband SE, Misfit Shine, Withings Pulse, Fitbit Zip, Omron HJ-203, Yamax Digiwalker SW-200 and Moves mobile application). In free-living conditions, 56 volunteers wore the same activity trackers for one working day. Test-retest reliability was analyzed with the Intraclass Correlation Coefficient (ICC). Validity was evaluated by comparing each tracker with the gold standard (Optogait system for laboratory and ActivPAL for free-living conditions), using paired samples t-tests, mean absolute percentage errors, correlations and Bland-Altman plots.

**Results:**

Test-retest analysis revealed high reliability for most trackers except for the Omron (ICC .14), Moves app (ICC .37) and Nike+ Fuelband (ICC .53). The mean absolute percentage errors of the trackers in laboratory and free-living conditions respectively, were: Lumoback (−0.2, −0.4), Fibit Flex (−5.7, 3.7), Jawbone Up (−1.0, 1.4), Nike+ Fuelband (−18, −24), Misfit Shine (0.2, 1.1), Withings Pulse (−0.5, −7.9), Fitbit Zip (−0.3, 1.2), Omron (2.5, −0.4), Digiwalker (−1.2, −5.9), and Moves app (9.6, −37.6). Bland-Altman plots demonstrated that the limits of agreement varied from 46 steps (Fitbit Zip) to 2422 steps (Nike+ Fuelband) in the laboratory condition, and 866 steps (Fitbit Zip) to 5150 steps (Moves app) in the free-living condition.

**Conclusion:**

The reliability and validity of most trackers for measuring step count is good. The Fitbit Zip is the most valid whereas the reliability and validity of the Nike+ Fuelband is low.

## Background

Activity trackers are developed to increase an individual’s awareness about physical activity behavior throughout the day. It is well known that regular physical activity decreases the risk of many chronic diseases and can improve quality of life [1–3]. A commonly used physical activity guideline is the 10,000 steps/day norm: healthy adults are recommended to take 10,000 steps per day to maintain physical fitness and health [4]. However, many people worldwide are not aware if they comply with this recommendation [1]. In addition, previous research has indicated that most people tend to overestimate their level of physical activity [5, 6]. Activity trackers may potentially overcome this issue.

Over the past five to ten years, an increasing number and variety of activity trackers have become available on the consumer market. Activity trackers are small and user friendly devices that measure the number of steps taken and/or the amount of time spent performing physical activities at different intensities. Most activity trackers also convert the number of steps with algorithms into measures such as the distance covered and the number of calories burned. Associated (mobile) applications provide users with insight into their individual physical activity behavior over a certain period of time. This might work as a motivator to increase physical activity [7, 8]. Consumer activity trackers might also be beneficial for scientific research, due to their ease of usability and relatively low cost. Examples of popular devices are the Fitbit, Jawbone Up, and Withings Pulse.

For accurate measurement and interpretation of the data, these devices must be reliable and valid. A number of studies have examined consumer tracker accuracy [6, 9–18], however, six studies were based upon earlier versions of Fitbit devices, and the methodology for assessing reliability and validity varied considerably. For example, different types of activity were used (walking on a treadmill at different speeds, lab cycling, walking stairs, daily activities), and different gold standards were utilized (energy expenditure [EE] measured by breath-to-breath analysis, self-reported physical activity translated to EE [in METs], and real step count). Five studies were performed in a laboratory condition [9–11, 14, 16], and six studies examined the reliability or validity of activity trackers during (semi-structured) free-living conditions [6, 12, 13, 15, 17, 18]. The validity of activity trackers may differ in free-living conditions compared to standardized lab conditions because of the increased variety in walking speeds, directions, intensities, etc. in free-living. To date, no studies have assessed reliability and validity of consumer trackers in both laboratory and free-living conditions. The aim of this study was to determine the reliability and validity of ten consumer activity trackers, in both a standardized laboratory condition and in free-living conditions.

## Methods

### Study design

The following ten activity trackers were examined: the Lumoback, Fitbit Flex, Nike+ Fuelband SE, Jawbone Up, Misfit Shine, Withings Pulse, Fitbit Zip, Omron HJ-203, Yamax Digiwalker SW-200 and the Moves mobile application. The Optogait system *(OPTOGait, Microgate S.r.I, Italy, 2010)* was used as the gold standard on the treadmill in the laboratory condition. This system consists of two beams attached to the sides of the treadmill. The system uses an LED lighting system to precisely measure the number of steps which is a reliable and valid method for measuring step count (cadence) [19]. The ActivPAL *(PAL Technologies Ltd., Glasgow, UK)* was used as the gold standard in the free-living condition. The ActivPAL was worn on the thigh underneath the clothing. Previous research has demonstrated that the ActivPAL is a reliable and valid tool for measuring the number of steps taken both on a treadmill and in free-living conditions [20–22].

### Study sample

Only healthy adult volunteers (age ≥18, <65 years) were included in the study. Participants were recruited through advertisements within the Hanze University and by using the individual networks of the researchers. Subscribers were excluded from participation if they experienced problems with standing or normal ambulation as well as if they performed daily activities which could possibly damage the activity trackers while being worn (when participating in the free-living study). All components of the study are described below in more detail. The study was in accordance with the principles as outlined in the Declaration of Helsinki and an exemption was obtained by the Medical Ethical Committee of the University Medical Center of Groningen for a comprehensive application. All participants were informed about the study procedures and provided informed consent prior to the initiation of this study.

### Testing under laboratory conditions

In order to examine the test-retest reliability and the validity of the ten trackers in a standardized situation, the participants walked for 30 min on a treadmill at a walking speed of 4.8 km/h. This walking velocity was similar to velocities used in previous treadmill studies and is based on an average walking speed [14, 23]. During the treadmill test, the participants wore all ten activity trackers and the ActivPAL. The Optogait system on the treadmill was used as the gold standard. The primary outcome measure was the total number of steps measured within the duration of the 30 min treadmill test. All participants repeated this test one week later.

### Testing under free-living conditions

In order to examine the validity of the ten trackers in free-living conditions during a working day, the activity behavior of the participants was measured during one working day between 9.00 am and 4:30 pm. The participants wore each ten different trackers and the ActivPAL simultaneously. During the specified day, participants performed their normal daily activities; however, they were requested to abstain from cycling or driving a vehicle during the test period. This was required in order to be able to make a realistic comparison between the trackers; because the different wearing positions of the trackers might influence step measurements during these activities. The primary outcome measure was the total number of steps measured between 9 am to 4:30 pm.

### Activity trackers

All devices utilized in this study are able to track step count.

#### Lumoback

The Lumoback™ *(Lumo BodyTech, Inc. Palo Alto, California, USA)* was worn around the lower back and was calibrated to the user by utilizing the associated application.

#### Fitbit Flex

The Fitbit Flex™ *(Fitbit, Inc., San Francisco, CA, USA*) is a wrist-worn tri-axial accelerometer and was worn on the non dominant arm.

#### Jawbone UP

The Jawbone UP™ *(JAWBONE, San Francisco, CA, USA,* is a wrist-worn three-dimensional activity tracker and was worn on the non dominant arm.

#### Nike+ Fuelband

The Nike+ Fuelband SE ™ *(Nike Inc., Beaverton, OR, USA)* is a wrist-worn three-dimensional activity tracker and was worn on the non dominant arm.

#### Misfit Shine

The Misfit Shine™ (*Misfit Wearables, Burlingame, California, USA)* is a small tri-axial accelerometer which was carried in the front pocket of the trousers.

#### Pulse

The Withings Pulse™ *(Withings, Issy les Moulineaux, France)* is a small tri-axial accelerometer which was carried in the front pocket of the trousers.

#### Fitbit Zip

The Fitbit Zip™ *(Fitbit, Inc., San Francisco, CA, USA)* is a small tri-axial accelerometer which was carried in the front pocket of the trousers.

#### Omron

The Omron Walking Style III™ (type HJ-203) *(OMRON Healthcare Europe B.V., Hoofddorp, the Netherlands)* is a pedometer with a two-dimensional sensor which was carried in the front pocket of the trousers.

#### Digiwalker

The Yamax Digiwalker SW-200™ *(YAMAX Health & Sports, Inc. San Antonio, USA, $39.50)* is a two-dimensional pedometer that was attached to the participant’s waistband.

#### Moves

The Moves^R^ is a smartphone application. It uses acceleration sensors from a smartphone and GPS to measure the number of steps taken. The mobile phone used in the laboratory study was an Iphone 4S *(Iphone 4S, Apple Inc., USA).* During the free-living study the smartphone of the participant was used (IOS/Android) and carried in the front pocket of the trousers.

### Statistical analysis

A sample size analysis was conducted to calculate the number of required participants. As previous data on relevant differences for sample size calculation does not exist, we reasoned that a difference of 10 % for the laboratory condition and 15 % for the free-living condition seemed appropriate. Using these relevant differences and expected mean number of steps in both conditions, it was calculated that at least 24 participants were necessary for participation in the laboratory condition and 58 participants for the free-living condition to enable substantiation of a relevant difference between the trackers and the gold standards with a power of 80 % and a significance level of 5 %. This number of participants is comparable to other validation studies [12, 14, 15]. This reassured our reasoned choice for using 10 % and 15 % as cut-off points for the mean difference.

Descriptive statistics were used to characterize the sample. Normality of the outcome measures was tested by Shapiro Wilk for all activity trackers in both parts of the study. Test-retest reliability of the trackers in the laboratory study was assessed by calculating the Intraclass Correlation Coefficient (ICC) (two-way random, absolute agreement, single measures with a 95 % confidence interval). Common cut-off points for reliability assessment were used; >.90 (excellent), .75-.90 (good), .60-.75 (moderate), and < .60 (low) [24].

The validity of the ten trackers was determined by several statistical tests. First, systematic differences between the activity trackers and the gold standards were assessed by the paired samples *t*-test. In the event of non-normally distributed data, the Wilcoxon Signed Rank test was used. Mean absolute percentage errors (c) compared to the gold standards were calculated with the following formula: mean difference activity tracker-gold standard x 100 / mean gold standard. Second, in order to examine the correlation between the trackers and the gold standards, the ICC was calculated (absolute agreement, two-way random, single measures, 95 % confidence interval). Third, to examine the level of agreement between the trackers and the gold standard, Bland-Altman plots were constructed with their associated limits of agreement. In addition, the ActivPAL scores from the laboratory study were compared with the corresponding Optogait scores by use of the three previously mentioned statistical tests, in order to assess the degree of consensus between the two gold standards used in this study.

## Results

For the laboratory study, 33 participants were included (16 males, mean age (±SD) 39 (±13.1) years, mean BMI (±SD) 23.6 (±2.2) kg/m^2^, and 17 females, mean age (±SD) 35 (±11.2), mean BMI 22.5 (±2.1) kg/m^2^). Thirty of the 33 participants performed the test again one week later. Most individuals who participated in the laboratory study also participated in the free-living study (*N* = 23) wherein a total of 56 participants were included (18 males, mean age (±SD) 37.1 (±10.6), mean BMI (±SD) 24.1 (±2) kg/m^2^, and 38 females, mean age (±SD) 30 (±9.5) years, mean BMI (±SD) 23.1 (±2.5) kg/m^2^). Most of the participants were university employees, with an office job. Activities performed by the participants during the test day included sitting (e.g., at the computer), standing (e.g., teaching activities) and walking. A number of participants were highly active (e.g., took a long walk during lunch time) whereas others were mainly sedentary during the test day. The Nike+ Fuelband and Moves app were tested with a fewer number of participants in the free-living study (*N* = 20 and *N* = 11 respectively). The Nike+ Fuelband was not available at the beginning but was included during the study. The Moves app was unavailable at no cost for most participants in the free-living study. In all 11 cases, the Moves app was operating on an Android device.

### Descriptive statistics

**Fig. 1:**
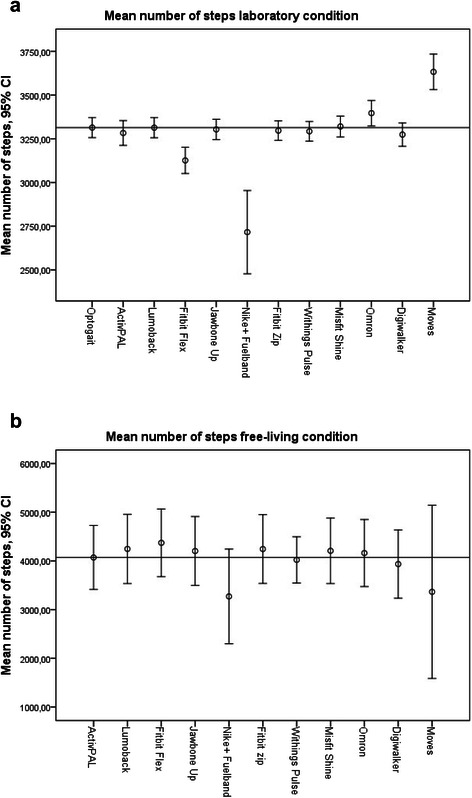
Descriptive Statistics (mean number of steps, 95 % CI) as measured by the gold standards (horizontal lines) and the ten activity trackers in the laboratory and free-living condition

Figure 1 depicts the descriptive statistics (mean number of steps, 95 % CI) as measured by the gold standards and by the ten activity trackers in both the laboratory (A) and free-living condition (B). The mean number of steps (±SD) measured by the Optogait in the laboratory condition was 3314 (±162), and the mean number of steps (±SD) measured by the ten trackers ranged from 2716 (±672) [Nike+ Fuelband] to 3633 (±286) [Moves app]. The mean number of steps (±SD) measured by the ActivPAL in the free-living condition was 4070 (±2430), and the mean number of steps (±SD) measured by the ten trackers ranged from 3271 (±2136) [Nike+ Fuelband] to 4372 (±2562) [Fitbit Flex]. As shown in Fig. 1, the Nike+ Fuelband and Moves app provide a relatively large confidence interval for the mean number of steps in the free-living condition, which is partly due to a lower number of measurements of these devices. Therefore, additional power analyses were executed, which are shown below.

### Agreement between the two gold standards

The ActivPAL was compared with the Optogait in the laboratory condition using the same statistical tests that were used for the ten activity trackers. The ActivPAL demonstrated a mean difference of 9 ± 6 steps [0.3 %] with the Optogait (*P* < 0.001, *N* = 25). The effect size of this significant difference was calculated using Cohens effect size [25] and indicated an effect size of 0.02, which is negligibly small. The ICC between the ActivPAL and the Optogait is 1. The Bland-Altman plot revealed a difference between the lower and upper limit of agreement of 24 steps. These results indicate excellent agreement of the two gold standards used in this study.

### Test-retest reliability

The ICCs between the first test and the second test (one week later) in the laboratory condition varied between 0.14 and 0.96 (Table 1). The gold standards used in this study (Optogait and ActivPAL), demonstrated excellent test-retest reliability. Test-retest reliability of the Lumoback, Fitbit Zip, and Withings Pulse was excellent as well (i.e., ICC > .90). Test-retest reliability of the Jawbone Up, Fitbit Flex, and Misfit Shine was good (ICC .75 - .90); test-retest reliability of the Digiwalker was moderate (ICC .60 - .75); and test-retest reliability of the Nike+ Fuelband, Omron, and Moves app was low (ICC < 0.60).

**Table 1:** Intraclass correlation coefficients between Test 1 and Test 2 of the treadmill walking test (*N* = 30)

Activity tracker	Intraclass Correlation Coefficient	95 % confidence Interval
*Optogait*	0.92**	*0.85 –0.96*
*ActivPAL*	0.96**	*0.90 –0.99*
Lumoback	0.90**	0.79 – 0.95
Fitbit Flex	0.81**	0.64 –0.91
Jawbone UP	0.83**	0.66 –0.91
Nike+ Fuelband	0.53**	0.22 –0.75
Misfit Shine	0.86**	0.73 –0.93
Withings Pulse	0.92**	0.83 –0.96
Fitbit Zip	0.90**	0.80 –0.95
Omron	0.14	−0.24 –0.47
Digiwalker	0.71**	0.47 –0.86
Moves app	0.37*	0.02 –0.64

*P<0.05

**P <0.01

### Systematic differences and mean absolute percentage error

In the laboratory condition, there was a significant difference between the number of steps measured by the Optogait (gold standard) and those measured by the Lumoback, Fitbit Flex, Nike+ Fuelband, Withings Pulse, Fitbit Zip, Omron, and the Moves app (Table 2). However, the size of the mean difference was less than 34 steps (MAPE = 1 %) or close to this MAPE for most of the trackers. There was a more substantial MAPE between the Optogait and Fitbit Flex; (188 steps [5.7 %]), the Moves app (319 steps [9.6 %]), and the Nike+ Fuelband (598 steps [18 %]). The Misfit Shine demonstrated the smallest MAPE compared with the Optogait [i.e., 0.18 %].

**Table 2:** Mean difference scores (gold standard – activity tracker) and MAPE in the laboratory and free-living condition

Laboratory Condition ^1^						Free-living condition ^2^				
	N	Mean difference (SD) ^a^	MAPE ^b^	t-value/Z-value ^c^	P-value	N	Mean difference ^a^	MAPE ^b^	Z-value ^c^	P-Value
*ActivPAL*	*25*	*9 (6)*	*0.3*	*7.19*	0.000 *	55				
Lumoback	32	8 (20)	0.2	2.24	0.033 *	51	17	0.4	−0.97	0.332
Fitbit Flex	33	188 (219)	5.7	4.93	0.000 *	54	−150	3.7	−2.23	0.026 *
Jawbone UP	32	34 (123)	1.0	1.60	0.119	53	−58	1.4	−0.24	0.851
Nike+ Fuelband	33	598 (618)	18.0	−4.36	0.000 *	20	977	24	−3.55	0.000 *
Misfit Shine	33	−6 (43)	0.2	−0.80	0.430	55	−43	1.1	−0.36	0.719
Withings Pulse	32	15 (23)	0.5	3.70	0.001 *	51	323	7.9	−5.24	0.000 *
Fitbit Zip	32	11 (12)	0.3	5.44	0.000 *	55	−49	1.2	−2.66	0.008 *
Omron	32	−82 (157)	2.5	−2.96	0.006 *	55	17	0.4	−0.71	0.479
Digiwalker	32	38 (145)	1.2	1.46	0.153	55	240	5.9	−2.04	0.041 *
Moves app	33	−319 (366)	9.6	−4.36	0.000 *	11	1529	37.6	−2.85	0.004 *

^1^Mean (±SD) Optogait = 3314 (±162) ^2^ Mean (±SD) ActivPAL = 4070(±2430) *significant p-value indicating a systematic difference of the activity tracker. ^a^positive values indicate an underestimation of the activity tracker and negative values indicate an overestimation. ^b^ MAPE = mean absolute percentage error ^c^In case of non-normality the Wilcoxon Signed Rank Test was used instead of the Paired Samples *T*-test

In the free-living condition, there was a significant difference in the number of steps between the ActivPAL (gold standard) and the Fitbit Flex, Nike+ Fuelband, Fitbit Zip, Withings Pulse, Digiwalker, and the Moves app (Table 2). Again, the MAPE values of the trackers were small (less than 10 %), except for the Nike+ Fuelband and the Moves app (24 % and 37.6 % respectively). The smallest MAPE values were between the ActivPAL and the Omron (0.4 %) and Lumoback (0.4 %). The power for the calculation of the Nike+ Fuelband and Moves app was 62 % and 39 %, respectively. The power for the remaining devices was high, i.e., greater than 99 %.

### Correlations

Table 3 illustrates the Intraclass Correlation Coefficients between the ten activity trackers and the gold standard, for both the laboratory study and the free-living study. In the laboratory study, the ICCs ranged from -.13 (Moves) to .99 (Lumoback, Withings Pulse, and Fitbit Zip). The ICCs in the free-living study ranged from 0.80 (Moves) to 1 (Fitbit Zip).

**Table 3:** Intraclass Correlation Coefficients between the activity trackers and gold standards in the laboratory and free–living study

	Laboratory study (*N* = 33) (Optogait)	95 % confidence interval	Free–living study (*N* = 56) (ActivPAL)	95 % confidence interval
*ActivPAL*	*1*	*0.94 – 1*		
Lumoback	0.99 **	0.98 – 0.99	0.99 **	0.98 – 0.99
Fitbit Flex	0.22 *	−0.08 – 0.5	0.96 **	0.94 – 0.98
Jawbone UP	0.98 **	.095 – 0.99	0.94 **	0.90 – 0.97
Nike+ Fuelband	0.12	−0.1 – 0.37	0.83 **	0.37 – 0.94
Misfit Shine	0.97 **	0.93 – 0.98	0.99 **	0.98 – 0.99
Withings Pulse	0.99 **	0.95 – 0.97	0.96 **	0.91 – 0.98
Fitbit Zip	0.99 **	0.96 – 0.99	1 **	0.99 – 1
Omron	0.59 **	0.27 – 0.78	0.98 **	0.96 – 0.99
Digiwalker	0.65 **	0.39 – 0.81	0.96 **	0.93 – 0.98
Moves app	−0.13	−0.32 – 0.15	0.80 **	0.05 – 0.99

*P<0.05 **P<0.01

### Level of agreement

**Fig. 2:**
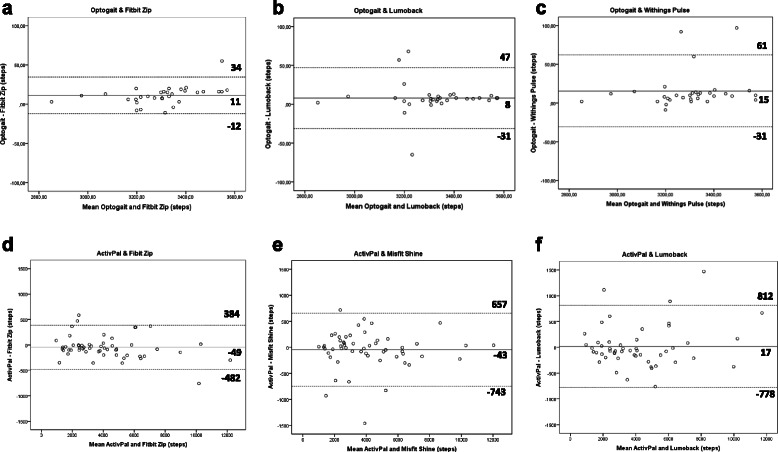
Bland-Altman plots of the top three activity trackers in the laboratory condition (Optogait vs. Fitbit Zip, Lumoback, and Withings Pulse, figure **a-c**) and free-living condition (ActivPal vs. Fitbit Zip, Misfit Shine, and Lumoback, figure **d-f**). The middle line shows the mean difference between the tracker and the gold standard, and the dashed lines indicate the limits of agreement (±1.96 * SD of the difference scores)

Bland-Altman plots indicate the differences between the tracker and the gold standard (y-axis) against the average of the two methods (x-axis). Table 4 indicates the mean differences with the gold standard and the limits of agreement for all activity trackers. In the laboratory condition, the plots showed the narrowest limits for the Fitbit Zip (46 steps), Lumoback (78 steps), and Withings Pulse (92 steps). The broadest limits were for the Nike+ Fuelband (2422 steps), Moves app (1436 steps), and Fitbit Flex (855 steps). In the free-living condition, the plots showed the narrowest limits for the Fitbit Zip (866 steps), Misfit Shine (1400 steps), and the Lumoback (1590 steps). The broadest limits of agreement were determined for the Moves app (5150 steps), Nike+ Fuelband (4528 steps), and Jawbone Up (3350 steps). Figure 2 illustrates the Bland-Altman plots for the top three activity trackers (narrowest limits of agreement) for both the laboratory (Fitbit Zip, Lumoback, and Withings Pulse) and for the free-living condition (Fitbit Zip, Misfit Shine and Lumoback).

**Table 4:** Mean difference scores with the gold standards and limits of agreement of the activity trackers in the laboratory and free-living study

	Mean difference (Optogait – tracker, lab study)^a^	Limits of Agreement		Mean difference (ActivPAL- tracker, free-living study)^a^	Limits of Agreement	
		Lower	Upper		Lower	Upper
*ActivPAL*	*9*	*−3*	*21*			
Lumoback	8	−31	47	17	−778	812
Fitbit Flex	188	−240	615	−150	−1424	1124
Jawbone UP	34	−54	81	−58	−1732	1618
Nike+ Fuelband	598	−613	1809	977	−1288	3240
Misfit Shine	−6	−91	85	−43	−743	657
Withings Pulse	15	−31	61	323	−864	1510
Fitbit Zip	11	−12	34	−49	−482	384
Omron	−82	−390	226	17	−1006	1040
Digiwalker	38	−248	323	240	−1028	1508
Moves app	−319	−1037	399	1529	−1046	4104

^a^ Positive values indicate an underestimation of the activity tracker and negative values indicate an overestimation

## Discussion

Ten popular consumer activity trackers were tested for their reliability and validity for measuring step count. Seven out of ten trackers were reliable (Lumoback, Fitbit Flex, Jawbone UP, Misfit Shine, Withings Pulse, Fitbit Zip, and Digiwalker), and five of these trackers also demonstrated high validity in laboratory conditions (Lumoback, Jawbone Up, Misfit Shine, Withings Pulse, and Fitbit Zip). The Moves app and Nike+ Fuelband exhibited low reliability and a low validity in laboratory conditions. In free-living conditions, the Fitbit Zip showed the highest validity and the Nike+ Fuelband indicated a low validity.

The validity of the ten activity trackers in laboratory conditions was examined with three methods of which the first was to assess systematic differences. According to Tudor-Locke et al. [23], activity monitors should not exceed a 1 % error deviation (MAPE) from the gold standard during walking on a treadmill at a speed of 3 mph (4.8 km/h) in order to be considered accurate. In the controlled lab-condition, five trackers achieved this condition: the Lumoback, Jawbone Up, Misfit Shine, Withings Pulse, and Fitbit Zip. The Digiwalker and Omron had an error deviation slightly higher than the 1 % threshold, e.g., 1.2 % and 2.5 %, respectively, which still represents a very low MAPE. The Fitbit Flex (5.6 %), Moves app (9.6 %) and Nike+ Fuelband (18 %) exhibited greater deviation errors whereby the Fitbit Flex and Nike+ Fuelband underestimated the number of steps, and the Moves app overestimated the number of steps. Some trackers were examined in other studies as well for systematic differences using comparable conditions. Melanson et al. [26] found an accuracy of 97.8 % of the Digiwalker SW-200 during walking on the treadmill with speeds between 3.0 and 3.5 mph (4.8 – 5.6 km/h), which is in accordance with our finding of 1.2 % error. In the study of De Cocker et al. [27], the Omron differed on an average of 6.7 % compared to the gold standard. The slightly smaller difference of 2.5 % determined in our study could possibly be explained by the longer duration of the treadmill test in this study (30 min vs. 5 min) which decreases the relative size of measurement error. Case et al. [16] found an error of +6.2 % for the Moves app installed on an IOS device and an error of −6.7 % for the Moves app installed on an Android device. The MAPE found for the IOS device was a bit lower than the +9.6 % difference in our study. An explanation could be the different version of the Iphone that was utilized (Iphone 5S compared to the 4S in our study). For the Nike+ Fuelband, Case et al. found a mean underestimation of 22.7 %. This was in line with our finding of 18 % underestimation.

The second method to determine validity was to examine the ICCs between the trackers and the gold standard. In the laboratory study, all trackers demonstrated a good to excellent agreement with the gold standard, with the exception of the Moves app, Nike+ Fuelband, and Fitbit Flex. Two other studies also examined correlations between the activity trackers and the gold standard in laboratory conditions. For the Fitbit One, Tacacs et al. [14] ascertained concordance correlations between 0.97 and 1.0 for five different speeds on the treadmill with manual steps counting as the gold standard. This was in accordance with our finding for the Fitbit Zip (ICC .99). For the Digiwalker SW-200, Beets et al. determined an ICC of .99 compared to real step count for children walking on a treadmill at the same speed (4.8 km/h) [28]. This is somewhat higher than the ICC found in our study (ICC .65). However, if we removed the four outliers in our analyses our ICC increased to .94, which is more in line with the findings of Beets et al.

The third and last way to examine validity was to assess the level of agreement by visualizing the data with Bland-Altman plots [29]. The difference between the lower and upper limit of agreement (Mean difference ± 1.96SD of difference scores) ranged from 46 steps (Fitbit Zip) to 2422 steps (Nike+ Fuelband). The Lumoback, Jawbone Up, Misfit Shine, Withings Pulse, and Fitbit Zip indicated the narrowest limits of agreement (less than 300 steps) which equals less than 10 % and less than 3 min walking. This can be considered as a relatively small range. Taken together with the small systematic differences of these trackers (less than 1 %), it is suggested that the Lumoback, Jawbone Up, Misfit Shine, Withings Pulse, and Fitbit Zip can be used interchangeably with the gold standard when walking on a treadmill. The systematic differences and the range between the upper and lower limits of agreement of the Moves app (1436 steps) and the Nike+ Fuelband (2422 steps) are considered to be too large to be used interchangeably with the gold standard.

To summarize, the lab results show that most trackers are valid with the Lumoback, Jawbone Up, Misfit Shine, Withings Pulse, and Fitbit Zip demonstrating the highest validity. The Moves app and Nike+ Fuelband are clearly invalid. It should be noted that, in a controlled lab condition, there is no variation in walking speed, intensity, direction, etc. which is in contrast to real life. Therefore, validity was also tested in free-living conditions.

The first way to validate activity trackers in free-living conditions was to assess systematic differences. In free-living conditions, an acceptable mean deviation from the gold standard is 10 % [23]. Eight activity trackers achieved this criterion. The Nike+ Fuelband and Moves app showed larger percentages of underestimation: 24.0 % and 37.6 %, respectively. Lee et al. [12] investigated various consumer trackers during different semi-structured activities (the participants followed a 69-min protocol), and compared total energy expenditure with the gold standard (breath-to-breath analysis). The Fitbit Zip, Jawbone Up, and Nike+ Fuelband differed 10.1 %, 12.2 %, and 13.0 %, respectively, from the gold standard. The differences are greater for the Fitbit Zip and Jawbone Up compared to the results of our study which could possibly be explained by the different outcome measure that was utilized in the study of Lee et al. (energy expenditure vs. step count). The difference between the Nike+ Fuelband and the gold standard is smaller compared to the present study (24 %). However, Lee et al. has already mentioned inconsistent results for the Nike+ Fuelband (a relatively small MAPE but also a low correlation with the gold standard) and, therefore, advised interpreting these results with caution. Ferguson et al. [17] investigated five similar devices (Jawbone UP, Nike+ Fuelband, Misfit Shine, Withings Pulse and Fitbit Zip) in free-living conditions for 48 h. They ascertained differences of 8.1 %, 25.6 %, 10.1 %, 6.3 % and 4.3 %, respectively. These values are in line with our findings in which the somewhat larger differences can be explained by the longer period of measurement. De Cocker et al., [27] investigated the Omron during free-living conditions and used the Digiwalker as a criterion measure. They reported a more substantial difference between the two devices compared to the findings of the present study (36.9 % vs. 0.4 %) which can be a result of non-walking activities, a longer period of measurement, and the different gold standard.

The second way to determine the validity of the activity trackers during free-living conditions was to calculate ICCs. All activity trackers were highly correlated to the gold standard (ActivPAL). The Nike+ Fuelband and the Moves app showed ICCs which were a bit lower and had broad confidence intervals (.83 [CI .37; .94] and .80 [CI .05 – .99] respectively). The high ICCs in the free-living study can be partially attributed to the differences in activity patterns between the participants during the test day; more variation increases the chances of a high ICC. Lee et al. [12] indicated similar results for the Fitbit Zip, Jawbone Up, and the Nike+ Fuelband, i.e., high correlations for the Fitbit Zip and Jawbone Up and a lower correlation for the Nike+ Fuelband. Tully et al. investigated the validity of the Fitbit Zip in free-living conditions; the Fitbit Zip was worn for seven days along with the Actigraph accelerometer. They reported a high correlation (Spearman Rho = .91) between steps/day when measured by the Fitbit Zip and by the Actigraph [15]. In addition, Ferguson et al. reported similar correlations for the Jawbone UP, Nike+ Fuelband, Misfit Shine, Withings Pulse, and Fitbit Zip in their free-living study of 48 h [17].

Finally, the level of agreement of the activity trackers with the gold standard during free-living conditions was assessed by Bland-Altman plots. The difference between the lower and upper limit of agreement ranged from 861 steps (Fitbit Zip) to 5150 steps (Moves app). For the Fitbit Zip, the range of 861steps (less than 1000 steps, e.g., 10 min walking) appears to be sufficiently low enough to be a valid measure in scientific research. The Misfit Shine and Lumoback demonstrated slightly larger limits of agreement (1400 and 1590 steps, respectively) which still demonstrates a good validity. For the other trackers, the limits of agreement show that, despite the relatively small systematic error (below 400 steps [10 %] for eight of the ten trackers), larger individual differences are evident, resulting in a lower validity.

To summarize, the validity of eight of the ten trackers was good during free-living conditions whereby the Fitbit Zip showed the best validity. The validity of the Nike+ Fuelband is low for measuring steps in free-living conditions.

Our study has some limitations. First, in the laboratory condition, only one type of activity was examined (walking), however, activity trackers can possibly perform differently during different activities or velocities (such as walking slow). The advantage of the 30-min measurement was that reliable data for average walking speed was obtained. Second, for examining free-living activity, we used a time span of 9:00–16:30 in which ‘occupational activity’ was mostly measured. The advantage of this method was that we were able to make a realistic comparison between the different trackers with different wearing positions because cycling was excluded. Cycling could have biased the results between centrally worn and wrist-worn trackers. However, the trackers might perform differently during a greater variety of activities such as more intensive exercise. These activities were not measured in this study. The third limitation was, that in the free-living condition, the Nike+ Fuelband and Moves app were tested with fewer number of participants. Because of a reasonable power (62 %), consistent results with the laboratory condition, and consistent results with other studies [12, 16, 17], the results of the Nike+ Fuelband are considered reliable. For the Moves app, only preliminary conclusions can be drawn on the validity in free-living conditions. This is due to the low N, consequently a lower power of 39 %, and because the Moves app was tested on different types of phones compared to the laboratory study (Android vs. IOS devices). Therefore, the results of the free-living condition cannot be compared with the lab condition because the different types of firmware may have influenced the results. However, our results for the Moves app on the different types of phones are comparable with the study of Case et al. [16] who showed that Android devices are associated with a modest underestimation, and IOS devices show a modest overestimation of step counting, which is in line with our results.

By combining the results of both conditions, it can be concluded that the validity of most activity trackers is good (Fitbit Zip, followed by Misfit Shine and Lumoback) or acceptable (Fitbit Flex, Jawbone Up, Withings Pulse, Omron, and Digiwalker). Looking at the wearing position of the trackers (wrist-worn for the Fitbit Flex, Jawbone UP, and Nike+ Fuelband and centrally worn, e.g. close to the pelvis or trunk, for the remaining devices), our results indicate that activity trackers worn close to the body exhibit a better validity than the wrist-worn activity trackers, especially during free-living conditions. For wrist-worn activity trackers, more measurement error can occur due to more variation in the way the arms are used in free-living conditions. This finding is supported by the research of Atallah et al. [30].

For the choice of a device, different considerations can be taken into account. First, the goal of physical activity measurement should be considered. For individual users, it is most important that the change in physical activity is clearly displayed, therefore, devices should be reliable. For large-scale research, the validity of a tracker is important in order to be able to compare physical activity levels of different groups. In addition, the type of activity that will be measured should be considered so a choice for the wearing position can be made. For example, wrist-worn activity trackers are better able to measure higher limb activity, and ankle worn trackers are better able to measure lower limb activity (e.g. cycling) [31]. Furthermore, a consumer can choose between a more advanced -and mostly more expensive device-, or a more simple and affordable device. This study demonstrated that less expensive devices are not necessarily less valid.

## Conclusions

In conclusion, the reliability of the Lumoback, Fitbit Flex, Jawbone UP, Misfit Shine, Withings Pulse, Fitbit Zip, and Digiwalker is good. These trackers are suitable for consumer usage and health enhancing programs. Of all ten trackers the Fitbit Zip shows the highest validity whereas the Nike+ Fuelband shows the lowest validity. The results of this study can assist consumers, researchers, and health care providers to make an evidence based choice for an activity tracker to measure step count.

## Abbreviations

EE: Energy expenditure
MET: Metabolic equivalent
BMI: Body mass index
km/h: Kilometers per hour
MAPE: Mean absolute percentage error
ICC: Intraclass correlation coefficient
CI: Confidence interval
